# Prescription Department

**Published:** 1892-05

**Authors:** 


LAXATIVE FOR CHILDREN.—

Steains' Cascara Arom'c ½ fl. oz.

Essence of Peppermint 1 fl. dr.

Syr. Senna 3½ fl. oz.

Mix. Sig.: One to two teaspoonfuls at bedtime.

SUB-ACUTE BRONCHITIS.—

℞ Ammon. chloridi, oz. ss.
Potass. chlorat, dr. iss.
Mist. glyc. comp., oz. iv.

M. Sig. Teaspoonful four times a day.—*Ex.*

HEMORRHAGE IN TYPHOID FEVER.—

℞ Pulv. opii, gr. x.
Ergotinnine citrate, gr. 1-10.
Pulv. plumbi acetas, gr. x.

M. fiat cap. No. x. Sig. One every four hours, oftener if necessary.—*Ex.*

INFANTILE DIARRHŒA.—

℞ Aq. calcis, oz. vj.
Syr. rhei arom., oz. j.
Tr. opii camph., oz. ss.
Tr. cardem. co., oz. ss.

M. One teaspoonful often to children under one or two years, afflicted with sour stomach attended with diarrhœa, vomiting, etc.—*Memphis Med. Monthly.*

CHRONIC DIARRHŒA AND DYSENTERY.—

℞ Cupri sulph., gr. vj.
Myrrhæ, gr. xij.
Confect. roses, fl. dr. ij.

Fiat pil. No. xii. One every six hours.—*Ex.*

SUMMER DIARRHŒA.—For childrer one year of age:

℞ Zinci sulphocarbolat., gr. v.
Bismuth subnitr., gr. xv.
Pepsin saccharat, dr. ss.

M. Et. in chart. No. xv div. Sig. One powder every hour until stools become inodorous; then every two to four hours.—*Waugh, Times and Register.*

NEURALGIA.—

℞ Acetanilid, dr. iss.
Camphor monobro., gr. xviij.
Pulv. rhamnus pursh., gr. xij.
Pulv. zingiberis, gr. ix.

M. ft. chart. No. 18. Sig. One powder after meals. Attention should be paid to the hygiene of the patient. —*Ex.*

—For the *Anœmia* of a young girl fifteen years of age, Prof. Da Costa prescribed:—

℞ Sodii arsenit., gr. ¼

Ferri sulphat., gr. ij

Potassii carbonat., gr. ij.

M. One pill three times a day, the dose to be gradually increased.

Also a diet of fresh animal food, with green vegetables by forced feeding (four or five meals in the twenty-four hours), or if quantity is an objection, the freshly extracted juices of meat may be given. Red wines might also be given with advantage; and the patient should lead an outdoor life.—*Ex.*

For Diarrhœa.—

℞ Tinct. opii camph., f oz. j
Misturæ cretæ, f oz. iij
Ol menthæ pip., mx.

M. A teaspoonful for an adult every three or four hours until diarrhœa is checked.

Infantile Syphilis—.

R. Protiodide of mercury, gr. iii;
Lactate of iron, gr. viii;
Powdered sugar, dr. ii.

To be made into ten powders, one of which is given three times a day. —*L' Union Medicale*, No. 12, 1892.

Injection for Chronic Cystitis.—

Ultzmann recommends the following prescriptions in the treatment of this troublesome condition:

Crystallized carbolic acid, gr. xv;
Distilled water, oz. iiiss.

Dissolve, and mix with equal parts of hot water at the moment that the liquid is to be injected.

Or the following:

Boric acid, oz. ss;
Glycerin, oz. i;
Distilled water, oz. x.

Make a solution, and mix with equal parts of warm water at the moment of employment.

Either one of these solutions when warm may be used for washing out the bladder in cases of chronic cystitis. When the vesical secretion is catarrhal and has a bad odor, the following injection is useful:

R. Nitrite of amyl, gtt. v;
Distilled water, oz. iv.

Mix, and add a tablespoon of this solution in the quanity of water for a vesical injection

